# The Applications of Magnetic Particle Imaging: From Cell to Body

**DOI:** 10.3390/diagnostics10100800

**Published:** 2020-10-09

**Authors:** Xiao Han, Yang Li, Weifeng Liu, Xiaojun Chen, Zeyu Song, Xiaolin Wang, Yulin Deng, Xiaoying Tang, Zhenqi Jiang

**Affiliations:** School of Life Science, Institute of Engineering Medicine, Beijing Institute of Technology, Beijing 100081, China; 3120170646@bit.edu.cn (X.H.); leon@buybiogoods.com (Y.L.); breeze@bit.edu.cn (W.L.); 3120185688@bit.edu.cn (X.C.); 3220181090@bit.edu.cn (Z.S.); icecreamgao@163.com (X.W.); deng@bit.edu.cn (Y.D.); tangxiaoying@bit.edu.cn (X.T.)

**Keywords:** magnetic particle imaging, cell tracking, tumor imaging, hyperthermia, imaging tracer, molecular imaging, nanoparticles

## Abstract

Magnetic particle imaging (MPI) is a cutting-edge imaging technique that is attracting increasing attention. This novel technique collects signals from superparamagnetic nanoparticles as its imaging tracer. It has characteristics such as linear quantitativity, positive contrast, unlimited penetration, no radiation, and no background signal from surrounding tissue. These characteristics enable various medical applications. In this paper, we first introduce the development and imaging principles of MPI. Then, we discuss the current major applications of MPI by dividing them into four categories: cell tracking, blood pool imaging, tumor imaging, and visualized magnetic hyperthermia. Even though research on MPI is still in its infancy, we hope this discussion will promote interest in the applications of MPI and encourage the design of tracers tailored for MPI.

## 1. Introduction

Medical imaging has been increasingly involved in decision making in every aspect of disease treatment, including diagnosis, treatment, and prognosis [1]. Assistance from medical imaging techniques has enhanced the understanding of disease and helped clinicians to achieve precise treatment and, therefore, improve the quality of human life and probability of survival. For instance, cancer is among the most important causes of human death. The poor performance of treatment for cancer diagnosed at an advanced stage has urged the development of detection techniques such as multiple types of medical imaging methods [2].

Imaging methods play an essential role in the diagnosis and treatment of different stages of cancer, such as the tracing, analysis, and treatment of tumors. Commonly used imaging methods include radionuclide imaging, optical imaging, magnetic resonance imaging (MRI), computed tomography (CT), and ultrasound (US). These imaging methods are capable of visualizing lesions and, therefore, play a crucial role in guiding the protocols and processes of cancer treatment. The imaging methods can generally be divided into categories according to their imaging principles: techniques that directly image tissues and methods that locate and image regions of interest by detecting and imaging tracers.

The commonly used medical imaging techniques have advantages and disadvantages in different scenarios. In terms of imaging methods that use markers to label the region of interest, MRI using contrast agents offers strong depth penetration and high spatial resolution. However, in T_1_-weighted MRI, the contrast agents show poor sensitivity, and in T_2_-weighted MRI, the agents are difficult to distinguish from biological tissue [3,4,5,6,7]. Radionuclide imaging has high sensitivity and can achieve long-term tracing, but the long half-life of the tracers may involve the greater net dose to the patient or weaker signal to noise ratio [8].Additionally, this imaging method has low spatial resolution and requires the injection of radioactive tracers [9,10,11]. Optical imaging has remarkably high sensitivity, but its penetration depth is low because photons are easily absorbed and scattered when penetrating biological tissues; therefore, it is difficult to image deep biological tissues with this technique [12,13]. The contrast-enhanced CT has high resolution but relatively low sensitivity [14]. Furthermore, CT scanning exposes patients to ionizing radiation [14]. The contrast-enhanced US can achieve real time imaging, has no ionizing radiation, and is cost efficient for patients [15]. However, it has relatively low sensitivity and low resolution [16].

Magnetic particle imaging (MPI) is a cutting-edge medical imaging technique. The principle of this technique is to locate and quantify the tracers at the region of interest by directly scanning superparamagnetic iron oxide nanoparticles (SPIONs) as an imaging tracer. MPI scans the electronic moments of SPIONs, which is 22 million times more intense than the nuclear moments that detected by MRI [8]. In addition, MPI has the characteristics of linear quantitativity, high sensitivity, and high spatial resolution. The spatial resolution of MPI is approximately 1mm, and has the potential to reach more than 300 µm with the improvement with SPIONs, hardware, and pulse sequences [8]. MPI has a voxel size of approximately 1 mm3, which is less than pre-clinical ^1^HMRI but close to that of ^19^FMRI and Positron Emission Computed Tomography (PET) [17].Additionally, because the device directly scans the imaging tracer, it also has high tissue penetration and almost no background signal from biological tissue [8]. In addition, MPI uses SPIONs as imaging tracers, which enable long-term tracking and are metabolizable and nonradioactive. Moreover, no current research shows that the currently used commercialized tracer that is also suitable for MPI has a significant impact on human health, which indicates that MPI has great possibilities and potential in clinical use.

In 2005, Bernhard Gleich and Jürgen Weizenecker published a paper on nature, discussing the theory and principle of MPI [18]. The Philips Laboratories built the first MPI scanner in the same year. Since then, researchers from Philips Laboratories, University of California, Berkeley, and other institutions have since contributed to the development of hardware construction and image reconstruction of MPI. In 2012, Conolly and Goodwill from the University of California, Berkeley, built the MPI scanner based on the same imaging principle but different scanning techniques and reconstruction approaches. Other researchers from the United States, Japan, Italy, and other countries [19,20,21,22,23,24,25] have also researched different aspects of, and contributed to, its development. With continuous improvement of MPI, significant progress has also been made in the applications of MPI in various fields.

## 2. The Principle of Magnetic Particle Imaging

The imaging principle of MPI is based on the nonlinear magnetization of SPIONs immersed in an alternating external magnetic field. The nonlinear magnetic properties of SPIONs can be described by the Langevin function. When an alternating magnetic field (AMF) is applied to SPIONs, the magnetic moments of those particles that are not magnetically saturated will change with the alternation of the field, while those of particles in the saturated state will not.

To utilize the magnetic properties of SPIONs for imaging, the primary MPI system consists of three parts: the selection field, the drive field, and the receiving coil. The selection field is a time constant field with sufficient magnitude that is constructed of two homopolar magnetic fields, which possesses a field free region (FFR) in between. The drive field is an AMF to which the SPIONs in FFR respond. The response of SPIONs leads to the variation of magnetization. The receiving coil detects the change in magnetization.

MPI detects the electronic moment of SPIONs [8]. When SPIONs are exposed to selection field, all particles except those in the FFR are saturated. When drive field is added onto those particles simultaneously with the selection field, only the direction of the magnetic moments of SPIONs in the FFR changes with that of the surrounding field. All other SPIONs in the selection field outside FFR will not response to the drive field because they are in the state of saturation. Those changes of SPIONs in FFR cause the oscillation of the magnetization, which induces a signal in the receiving coil. Then, the FFR moves within the region of interest to generate MPI images, by assigning the signal to an image location according to the corresponding location of FFR [8]. The reconstruction of MPI image requires algorithms such as X-space [20,21,26,27,28,29,30,31,32,33].

## 3. MPI Tracers

MPI detects the tracer rather than its surrounding environment, such as blood or biological tissue. Hence, the performance of the tracer directly affects the imaging quality of MPI. Therefore, research on MPI tracers is essential for the development of MPI [24,34,35]. At present, the most commonly used MPI tracers, such as Resovist and Feraheme, are not tailored to the MPI physics. Moreover, fewer tracers are designed for MPI according to its imaging principle. In terms of MRI, studies have shown that the contrast can shorten the T2 relaxation time of MRI. These nanomagnetic substances will add magnetic micro-gradients onto the external magnetic fields, resulting in the loss of phase coherence of resonance protons, which leads to signal voids in the MR image [17]. In terms of MPI, the device directly images the SPIONs in the region of interest; therefore, it does not produce background signal except where SPIONs are concentrated. The differences in imaging principles between the two techniques lead to differences in tracer requirements. Considering the tracer behavior and imaging principle, characteristics such as size, shape, and magnetization saturation of the SPIONs affect the MPI signal. Therefore, exploring and designing tracers suitable for MPI is an essential direction in MPI research [34,36,37,38]. For example, Rao Hongyi’s research group at Stanford University used different iron oxide particles that were produced with a series of preparation times to explore the influence of preparation time on MPI tracers because those differences in time resulted in various iron oxides [3]. They demonstrated that Fe_3_O_4_ rather than Fe_2_O_3_ generated MPI signal. Jürgen Weizenecker et al. of the University of Applied Science discussed the parameters that affect the performance of MPI tracers by modeling the particle dynamics of it [39]. According to their research, parameters such as diameter, shape, and magnetization saturation affect the particle dynamics, thereby influencing the MPI signal [34,39]. Tian’s group from the Chinese Academy of Sciences and co-author controlled the shape and size of SPIONs to design a tracer tailored to MPI physics. They designed a cubic SPION with an edge length of 22 nm, which has high sensitivity and high resolution [40].

## 4. Applications of MPI

Research on MPI applications in various fields has made obvious progress in the development of MPI theory and instruments. Currently, because MPI can locate and quantify the imaging tracer and has no restrictions on penetration depth, its suggested applications mainly include multimodal imaging [3,22], cell tracing [3,40,41,42,43,44,45,46], inflammation tracing [47], drug delivery and monitoring [48,49], blood pool imaging [50,51,52,53], tumor detection [22,44,54,55], and arbitrary localization of magnetic hyperthermia [46,56,57,58,59]. In this review, we discuss four categories of applications in which most current MPI research studied: cell tracking, tumor detection, blood pool imaging, and hyperthermia.

### 4.1. Cell Tracking

Among the earliest applications of MPI was to use SPIONs to track cells, which is similar to MRI [60,61,62,63,64]. MPI has unique advantages in cell tracing. First, it has high sensitivity and resolution that enable the device to detect as few as 200 labeled cells [8]. In addition, a currently used imaging tracer is superparamagnetic nanoparticles, which are relatively small and can be easily uptaken by cells. Moreover, there are safe, biocompatible magnetic nanoparticle tracers—Feraheme—approved by the Food and Drug Administration (FDA) for clinical use. In addition, since MPI has high tissue penetration and no background noise, these characteristics make it suitable for in vivo cell tracking [42].

For example, Rao’s research team at Stanford University researched on MPI applications from in vitro to in vivo [3]. In this study, they designed a ferroferric oxide nanoparticle wrapped in a semiconductor polymer as an MPI tracer. This tracer had excellent performance in MPI scanning. Its MPI signal intensity is three times that of the commercial MPI imaging tracer VivoTrax and seven times that of the MRI tracer Feraheme. The semiconductor polymer coating material allowed the nanoparticles to be fluorescently imaged, thus enabling multimodal imaging, including MPI, MRI, and fluorescence imaging. The research team coincubated these superparamagnetic particles with the human cervical cancer cell line HeLa and then used MPI, MRI, and fluorescence imaging to scan the cells. The results showed that MPI had a better signal-to-noise ratio, and the intensity of the MPI signal was linearly related to the number of cells. In addition, MPI could detect a minimum of 250 labeled cells according to the imaging results. To investigate the application of MPI in vivo, researchers injected labeled HeLa cells into the abdomens of mice by subcutaneous injection. They then scanned the mice using MPI, MRI, and fluorescence imaging. According to the results, MPI could effectively track the labeled cancer cells injected into mice. In addition, because MPI directly imaged the particles rather than surrounding tissues, its penetration depth was not limited. Therefore, whether the device was scanning the mouse from the front or the back, the MPI signals had a high signal-to-noise ratio and contrast. The research team also conducted a long-term imaging study on mice, which showed that MPI signals could still be detected 10 to 20 days after the injection of cancer cells labeled with the particles. In addition, the signal was reduced by less than 20%, which demonstrated that MPI could achieve long-term cell tracking.

There are also other studies on the application of MPI in cell tracing. For example, Bo Zheng et al. of the University of California, Berkeley, used Resovist to label human mesenchymal stem cells (hMSCs). They then performed long-term tracking of the distribution of labeled stem cells that were injected into mice through the tail vein [42]. Pratx et al. of Stanford University used superparamagnetic particles to label exosomes released either by hypoxic tumor cells or by normal tumor cells. They used MPI to trace those labeled exosomes and showed that the exosomes released by hypoxic cancer cells were taken up more readily by hypoxic cancer cells. The exosomes released by hypoxic cancer cells can thus deliver drugs more effectively to the hypoxic area of the tumor [45].

**Figure 1:**
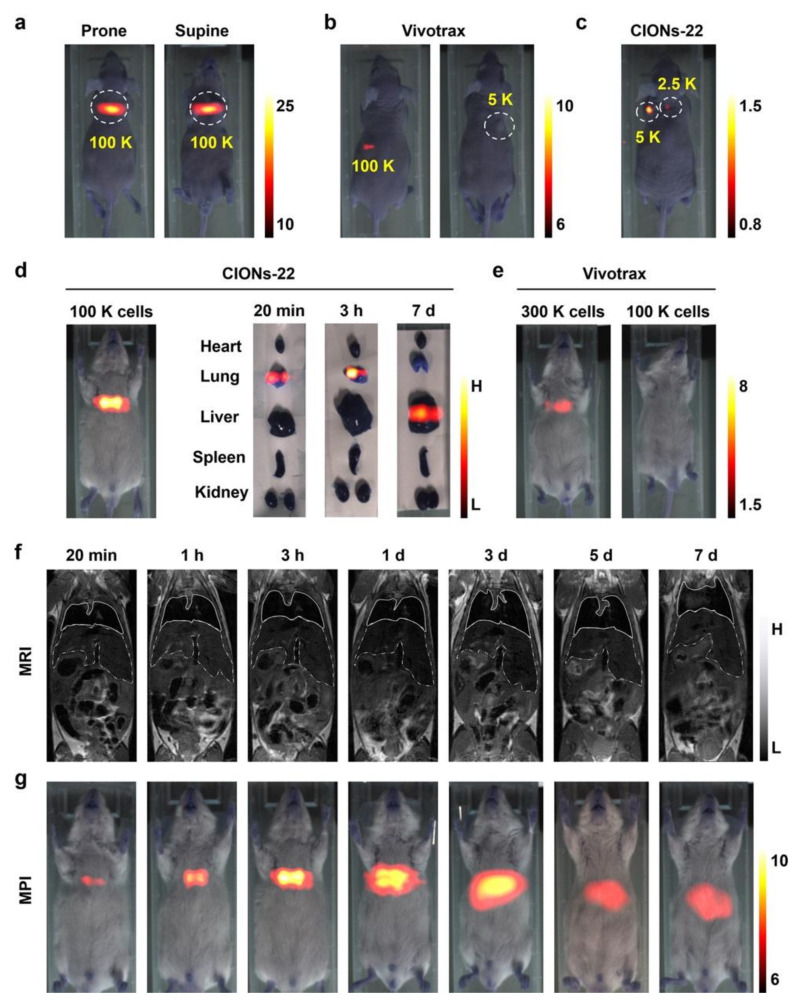
In vivo experiments that used MPI to track different numbers of stem cells injected subcutaneously (s.c.) into mice. The cells were labeled with the MPI-tailored tracer CIONs-22 or VivoTrax. The images are MPI images of a mouse (**a**) injected with 100 K stem cells labeled with CIONs-22, (**b**) injected with 100 K or 5 K stem cells labeled with VivoTrax, (**c**) injected with 5 K or 2.5 K stem cells labeled with CIONs-22, (**d**) 3 h after intravenous (i.v.) injection of 100 K CIONs-22-labeled stem cells and postmortem CIONs-22-labeled stem cell biodistribution 20 min, 3 h, or 7 days after i.v. administration, and (**e**) 3 h after the i.v. injection of 300 K or 100 K VivoTrax-labeled stem cells. (**f**) MRI images of a representative mouse after i.v. injection of 300 K CIONs-22-labeled stem cells. (**g**) Longitudinal 2D projection MPI images of a representative mouse within 1 week after i.v. injection of 100 K CIONs-22-labeled stem cells. Image reproduced, with permission, from Reference [40]. Copyright 2020 American Chemical Society.

Another example is the work of Tian’s group from the Chinese Academy of Sciences and co-author (Figure 1) [40]. They designed a specialized tracer tailored to MPI physics theory by controlling the size and shape of the particle. This tracer had high saturation magnetization and provided high sensitivity and high resolution in MPI. It performed remarkably well in the labeling and tracking of stem cells compared to the commercialized tracer VivoTrax. The tracer also achieved the real-time and long-term tracking of stem cells in vivo.

### 4.2. Tumor Detection

As cancer remains among the leading causes of human death, its diagnosis and treatment, especially early-stage diagnosis, have become among the most crucial research areas [8]. The cure rate of cancer strongly correlates with the stage of cancer that is diagnosed. If cancer can be diagnosed and provided with proper treatment at an early stage, the cure rate will increase significantly [2]. Research on devices with high sensitivity and high resolution is critical to the diagnosis of early-stage cancer. The high sensitivity of MPI is suitable for tumor detection because its characteristics satisfy the requirements of early-stage cancer diagnosis [8]. In practice, MPI takes advantage of the permeability and retention (EPR) effect to detect tumors. The EPR effect is caused by leaky vessels with large pores that enable the tumor to grow rapidly. This effect is commonly used to target tumors in the application of magnetic nanoparticles [38] and other nano contrast agents [8,65] in medical imaging. Therefore, MPI has great clinical application value in the early diagnosis of cancer. Moreover, experiments show that MPI can track tumors in vivo. Therefore, scientists from different countries have conducted various experiments and made significant progress in the application of MPI in tumor detection.

**Figure 2:**
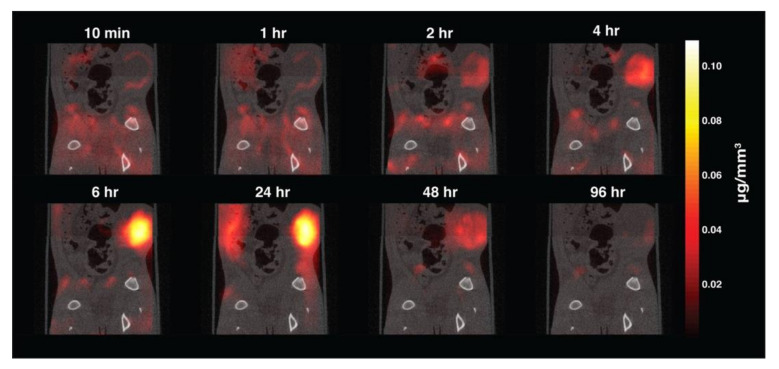
The imaging results of MPI volume coregistered to corresponding CT slices allow clear visualization of tracer dynamics in rats, including initial rim enhancement followed by accumulation and clearance. Image reproduced, with permission, from Reference [8]. Copyright 2017 American Chemical Society.

For example, the Conolly group of the University of California, Berkeley, explores the application of MPI to tumor detection (Figure 2). In their research, they used magnetic nanoparticles as imaging tracers because the particles accumulated in the tumor by the EPR effect [8]. The tracer was intravenously injected into the mice through the tail vein, and then a series of scanning time points was selected for tumor tracking. According to the MPI imaging results, the magnetic nanoparticles were first distributed to organs with larger blood volumes, such as the heart and lungs. As the intravascular signal decreased with time, the signal in the liver and kidney increased. In terms of tumor detection, the tumor rim enhancement caused by the initial vascular wash-in could be seen in detail through MPI scanning. Then, the scanning results showed that the magnetic nanoparticles began to accumulate inside the tumor due to the EPR effect. By 96 h after injection, the magnetic nanoparticles had cleared through metabolization.

In addition, recent studies have concentrated on the multimodality of medical imaging using MPI. Krishnan et al. of the University of Washington prepared a glioma-targeted MPI tracer by modifying iron oxide nanoparticles with lactoferrin-targeting brain tumors. They conducted in vivo imaging on tumor-bearing mice using a modified tracer [22]. According to the imaging results, the MPI signals of the tumor-bearing mice have high sensitivity and contrast.

The Jianghong Rao group of Stanford University designed a multimodality nanoparticle tracer that enabled multimodal imaging to detect tumor xenografts in living mice [65]. The imaging modalities included MPI, MRI, photoacoustic imaging, and fluorescent imaging. The tracer was composed of a Janus Fe_3_O_4_@semiconducting polymer nanostructure [3] and coated with semiconducting polymers that had high biocompatibility. According to the in vivo MPI results, the designed nanoparticles had slower reticuloendothelial system (RES) uptake and a significantly longer half-life in blood circulation and were more effective for passive tumor targeting via the EPR effect than VivoTrax. Multimodal imaging of orthotopic brain tumor xenografts in mice showed that both the MPI image and fluorescence image of the tumor were clear after injection of the MNPs, but the fluorescence image showed strong background signals. The T2-weighted MRI signal of the tumor was not obvious because of the negative contrast and lower sensitivity of iron oxides.

### 4.3. Blood Pool Imaging

MPI generates negligible tissue background signal and has a high signal-to-noise ratio. Because of the resulting high image contrast and high sensitivity, MPI has advantages in blood pool imaging in certain scenario compared with other imaging methods that use contrasts, such as CT and MRI [40]. For instance, among the main problems in blood pool imaging is to distinguish the blood in the capillaries from the surrounding tissues. Typically, in CT angiography (CTA) and MR angiography (MRA), a minor change in the background signal is indistinguishable from a slight change in imaging contrast in capillaries [66]. However, because MPI detects only the magnetic nanoparticles in the blood and does not image surrounding tissues that do not contain magnetic nanoparticles, the device will generate images that can more easily distinguish blood and the surrounding tissues. In conclusion, MPI has positive contrast, high sensitivity, high resolution, and no limitation on penetration depth, and it can achieve fast scanning and long-term tracking. These characteristics give MPI certain advantages and research value in the application of blood pool imaging. At present, MPI is used in the measurement of intestinal bleeding [51], brain injury [66], and cerebral blood volume [53] and has provided significant research results.

**Figure 3:**
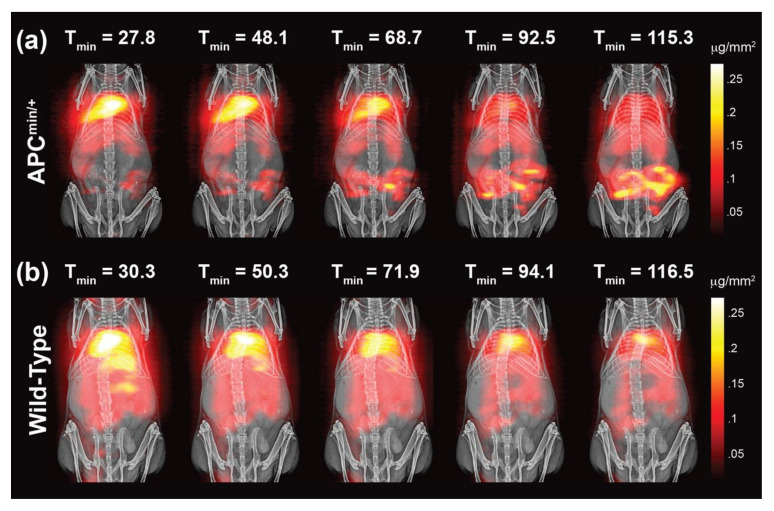
The imaging results of MPI coregistered with projection X-ray capture whole-body tracer biodistribution. (**a**) MPI shows the dynamics of tracer extravasation into the gut of an ApcMin/+ mouse over time. (**b**) No tracer extravasation into the gut is captured in the MPI images of a wild-type mouse over time. Image reproduced, with permission, from Reference [51]. Copyright 2017 American Chemical Society.

The Conolly group at the University of California, Berkeley, leveraged the high sensitivity, positive contrast, and linear quantitativity of MPI to detect gut bleeding in a murine model (Figure 3) [51]. Studies showed that MPI enabled the high contrast visualization of gastrointestinal (GI) bleeding in a GI polyp development mouse model according to the aggregation of magnetic nanoparticles. In addition, because the intensity of the MPI signal was linearly correlated with the mass of magnetic nanoparticles, the device could achieve gut bleed quantification using a nonradioactive and long circulating PEGylated tracer. The Conolly group also explored the application of MPI in imaging traumatic brain injury [66]. Research showed that MPI could detect the initial stage of traumatic brain injury and the corresponding hematoma in the closed skull. In addition, the device could be used to determine the location, severity, and depth of bleeding of any traumatic brain injury in the closed skull. Researchers such as Wald et al. of Massachusetts General Hospital used MPI to detect changes in cerebral blood volume by monitoring the concentration of SPIONs [53]. Studies have shown that MPI could detect hypercapnia at a high carrier-to-noise ratio.

### 4.4. Hyperthermia

Magnetic hyperthermia therapy kills cancer cells by heating tumors tissue. The approach includes delivering superparamagnetic nanoparticles to tumors and then adding a high frequency AMF that has no limitation on penetration depth. The particles respond to the AMF generate and release heat to the surrounding environment. However, if all particles within the AMF generate heat, surrounding healthy tissues may be damaged. Therefore, the particles that respond to the AMF need to be restricted in the targeting region. In addition, accurate dosage is needed to ensure that the tumor cells are killed without damaging the surrounding healthy tissues. Therefore, the precise targeting and dose control of magnetic hyperthermia are critical problems to be solved in clinical practice [67].

Because SPIONs can generate MPI signals and heat simultaneously, MPI can form a platform for precise real-time visualized magnetic hyperthermia based on those particles [58]. Precise heating location can be achieved if the nanoparticles are transferred to the tumor area and the hyperthermia treatment is limited to the treatment area. Therefore, MPI can achieve precision in magnetic hyperthermia because according to its imaging principle, only the particles within the FFR respond to the high frequency AMF and generate energy to heat surrounding tissue. In contrast, particles covered by the gradient field but outside the FFR are in a state of saturation and will not generate heat even within the AMF. Then, if the frequency of the AMF is intense enough, the magnetic nanoparticles within the FFR will be excited by the field and generate adequate thermal energy, thereby killing the cancer cells. The location of the hyperthermia treatment can be accurately manipulated by controlling the position of the FFR [58].

MPI can enable dose planning for hyperthermia. The MPI signal is linearly quantitative for the mass of the magnetic nanoparticles, and the specific absorption rate (SAR) is proportional to the mass of the magnetic nanoparticles. Therefore, the SAR image can be obtained from the MPI image. The MPI gradient field is used as an SAR filter to locate the spatial position of the treatment area accurately. Therefore, the SAR dose after gradient localization can be predicted [58].

**Figure 4:**
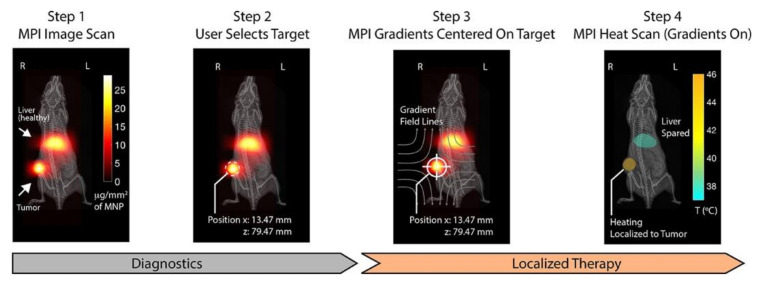
Theranostic workflow on a xenograft mouse model with SPIONs in its liver and tumor. Step 1: With an MPI scan at 20 kHz and 20 mT, the SPION present in the tumor and in healthy clearance organs (liver) can be seen clearly. The imaging parameters do not cause the SPIONs to generate heat. Step 2: The user selects a region, such as the tumor, to localize the magnetic hyperthermia. Step 3: The field free region (FFR) is located in the selected area by shifting the gradient field. This prevents the magnetically saturated SPIONs from generating heat. Step 4: A heat scan at 354 kHz and 13 mT is conducted with the MPI gradients held in position. Heating is experimentally restricted within the FFR while minimizing heat damage to the liver. Image reproduced, with permission, from Reference [58]. Copyright 2018 American Chemical Society.

Conolly et al. of the University of California, Berkeley, used different coils to change the frequency of the excitation field to achieve various purposes, such as diagnosis or treatment (Figure 4) [58]. In their research, the Conolly group demonstrated the application of MPI in hyperthermia and achieved dose planning and heat location. This idea was proven through a series of experiments, including in vitro experiments, in vivo experiments, and real-time temperature monitoring.

Yang Du from the Chinese Academy of Sciences and co-authors also works on applications of MPI in hyperthermia [59]. They designed a multimodal MPI/MRI tracer with high imaging performance and active biological targeting for magnetic hyperthermia therapy. This tracer distributes uniformly in the tumor when injected intratumorally and, therefore, provides superior performance in hyperthermia because it achieves uniform heating of the tumor.

## 5. Conclusions and Outlook

The applications of MPI still need further exploration. According to its imaging characteristics, researchers applied MPI to problems in the field that the existing technology cannot solve well, or to problems in which MPI can further improve the current methods. Researchers have also tried to merge MPI with other imaging techniques and used the advantages of different techniques to improve imaging quality comprehensively. At present, MPI has enabled significant progress in various areas, such as cell tracking, tumor imaging, drug delivery, blood pool imaging, and visualized magnetic hyperthermia. More applications of MPI in the fields of diagnosis and treatment should be explored, taking advantage of characteristics of MPI such as high resolution, high sensitivity, unlimited penetration depth, linear quantitativity, real-time imaging, and no radioactivity [52].

MPI scanners are currently not applied in the clinic. Both Wald and Magnetic Insight are trying to manufacture MPI instruments designed for the human body, but there are no finished products to date [5]. Moreover, tracers designed specifically for MPI also require further research. At present, the commonly used MPI tracers are contrast agents designed for MRI, such as Resovist (VivoTrax) and Feraheme [5]. Researchers are studying imaging tracers designed for multimodal imaging that include MPI and other imaging methods. However, among the crucial hindrances to the development of clinical MPI is that no tracer tailored to MPI physics is clinically approved [5]. Therefore, the development of MPI tracers for different diseases is a vital research direction for MPI development in the future.

Although MPI research is still in its infancy, researchers have tried to explore the possibilities of MPI in different fields. With the further development of MPI devices and tracers, the clinical application of MPI will be expanded. Therefore, further exploration of MPI applications in the future will also be a primary research direction in this field.
